# Microalbuminuria: A novel biomarker of sepsis

**DOI:** 10.4103/0972-5229.63034

**Published:** 2010

**Authors:** Surupa Basu, Mahuya Bhattacharya, Tapan K. Chatterjee, Subimal Chaudhuri, Subhash K. Todi, Arghya Majumdar

**Affiliations:** **From:** Department of Laboratory Medicine, Advanced Medicare Research Institute Hospitals, Kolkata, India; 1 **From:** Department of Critical Care, Advanced Medicare Research Institute Hospitals, Kolkata, India; 2 **From:** Department of Nephrology, Advanced Medicare Research Institute Hospitals, Kolkata, India; 3 **From:** Department of Pharmaceutical Technology, Jadavpur University, Kolkata, India

**Keywords:** Capillary permeability, critically ill, intensive care units, microalbuminuria, sepsis, mortality

## Abstract

**Context:**

Diffused endothelial dysfunction in sepsis leads to an increase in systemic capillary permeability, the renal component manifesting as microalbuminuria. The degree of microalbuminuria correlates with the severity of the acute insult, the quantification of which may serve to predict sepsis and mortality in critically ill patients.

**Aims:**

To evaluate whether the degree of microalbuminuria could differentiate patients with sepsis from those without and predict mortality in critically ill patients.

**Settings and Design:**

Prospective, non-interventional study in a 20-bed Intensive Care Unit (ICU) of a tertiary care hospital.

**Methods and Materials:**

After exclusions, between Jan-May 2007, 94 consecutive adult patients were found eligible. Albumin-creatinine ratio (ACR, mg/g) was measured in urine samples collected on ICU admission (ACR1) and at 24 hours (ACR2).

**Results:**

Patients were classified into two groups: those with sepsis, severe sepsis and septic shock (n = 30) and those without sepsis [patients without systemic inflammatory response syndrome (SIRS) and with SIRS due to noninfectious causes] (n = 64). In the sepsis group, median ACR1 [206.5 (IQR129.7-506.1)] was significantly higher compared to the non sepsis group [76.4 (IQR29-167.1)] (*P* = 0.0016, Mann Whitney). The receiver operating characteristics (ROC) curve analysis showed that at a cut off value 124 mg/g, ACR1 may be able to discriminate between patients with and without sepsis with a sensitivity of 80%, specificity of 64.1%, positive predictive value (PPV) of 51.1% and negative predictive value (NPV) of 87.3%. The median ACR2 [154 (IQR114.4-395.3)] was significantly higher (*P* = 0.004) in nonsurvivors (n = 13) as compared to survivors [50.8 (IQR 21.6-144.7)]. The ROC curve analysis revealed that ACR2 at a cut-off of 99.6 mg/g could predict ICU mortality with sensitivity of 85%, specificity of 68% with a NPV of 97% and PPV of 30%.

**Conclusion:**

Absence of significant microalbuminuria on ICU admission is unlikely to be associated with sepsis. At 24 hours, absence of elevated levels of microalbuminuria is strongly predictive of ICU survival, equivalent to the time-tested APACHE II scores.

## Introduction

Sepsis remains a major global healthcare concern, owing to high morbidity and mortality, despite the advances in medical therapeutics.[1,2] Targeted therapies probably lose their efficacy due to late administration.[3,4] Till date, there is no reliable method of diagnosing sepsis early in the critically ill.

Sepsis is marked by a severe host defense response that involves triggering of potent inflammatory cascades which release a plethora of pro-inflammatory molecules into the circulation.[5] The endothelium becomes dysfunctional due to the sustained onslaught of the inflammatory molecules and the simultaneous oxidative stress. An early event is the loss of barrier integrity leading to systemic capillary leak.[6] The glomerular manifestation of this enhanced capillary permeability is increased excretion of albumin in the urine.[7]

Microalbuminuria, defined as 30–300 mg/day of albumin excretion in the urine, occurs rapidly after an acute inflammatory insult such as sepsis and persists in patients with complications.[8–13] It is a common finding in critically ill patients, where it has shown promise not only as a predictor of organ failure and vasopressor requirement but of mortality, fairing better than Acute Physiological and Chronic Health Evaluation (APACHE) II score and Sequential Organ Function Assessment (SOFA) scores.[14–19]

Similar endothelial dysfunction may occur in nonseptic inflammatory states. But, it is not known whether the *degree* of microalbuminuria is different after a septic insult when compared to noninfectious ones such as pancreatitis, burns, trauma etc. and, whether it could delineate sepsis in a heterogeneous population of critically ill patients. By drawing an analogy with current biomarkers of sepsis such as procalcitonin (PCT), C-reactive protein (CRP) and the markers of endothelial damage such as the adhesion molecules, which are relatively elevated in sepsis;[20,21] we surmised a similar occurrence for microalbuminuria. To test this hypothesis, our study endeavored to explore a diagnostic role of microalbuminuria, by quantifying its level in patients with and without sepsis. A secondary aim was to evaluate the ability of microalbuminuria to predict mortality in the ICU.

## Materials and Methods

Consecutive adult patients admitted to a 20-bed mixed medical-surgical ICU in a tertiary care hospital were recruited between January and May 2007. The ethics committee waived formal informed consent, in view of the non-interventional nature of the study and approved the study protocol. All adult patients (> 18 years old) with ICU stay for more than 24 hours were included. It was determined *a priori* that patients would be excluded if they had anuria, macroscopic hematuria [confirmed with dipstick], history of preexisting chronic kidney disease (patients on long term renal replacement therapy and/or sonologic features of chronic damage and/or history of glomerular filtration rate of <30 ml/min). Female patients with menstruation or pregnancy were also excluded. Retrospectively, patients with significant proteinuria [more than 1+ protein on dipstick] due to renal and post renal causes, for example urinary tract infection, were excluded.

The study protocol was designed as per recommendations of the Standards for Reporting of Diagnostic Accuracy steering committee.[22]

On admission, the following data was collected for each patient: age; gender; date and time of admission; patient's clinical classification (medical or surgical), provisional diagnosis; co-morbid conditions such as diabetes, hypertension and chronic kidney disease. Clinical and laboratory data were collected; cultures sent and antibiotics administered within 24 hours of admission were noted.

APACHE II scores were calculated from data collected during the first 24 hours following ICU admission. Each patient was followed up for a maximum of 28 days and the following outcome data were obtained: ICU length of stay and ICU mortality.

At the time of admission and again after 24 hours, an intensivist examined patients for vital signs and symptoms of systemic inflammatory response syndrome (SIRS) and/or infection. Infection was delineated by presence of clinical signs and laboratory markers of inflammation along with presence of polymorphonuclear cells in a normally sterile body fluid and/or culture or gram stain of body fluids showing a pathogenic microorganism and/or radiological or visual evidence of an infective focus. The American College of Chest Physicians/Society of Critical Care Medicine Consensus Conference definitions were used to delineate patients with SIRS, sepsis, severe sepsis and septic shock.[23,24] On the basis of the above, patients were divided into two groups: patients *without sepsis* [i.e. no SIRS (patients without features of SIRS) and patients with SIRS due to noninfectious causes] and patients with *sepsis* [patients with SIRS and infection, including patients with severe sepsis and septic shock]. Patients who were found to have new infection after 48 hours of ICU admission were not included in the sepsis group as the infection was considered to be nosocomial.

Spot urine samples were collected by ICU nurses within 6 hours of admission and again at 24 hours, for quantification of ACR, which were referred to as ACR1 and ACR2 respectively. Urine samples were received in the biochemistry lab and stored at -20°C till analysis. Urinary microalbumin was measured by the immunoturbidimetric method and urinary creatinine by modified kinetic Jaffe reaction (Dimension RxL Max, Dade Behring Inc., U.S.A). The methods covered an analytical range of 1.3–100 mg/L for microalbumin and 0-20 mg/dl for creatinine. Microalbuminuria was defined by ACR values between 30 and 299 mg/g. ACR of > 300 mg/g is considered as clinical proteinuria. ACR < 30 mg/g is normal for a healthy population. These threshold values are well accepted for clinical use and have been predefined on the basis of published literature.

The intensivist who documented the clinical profile and the investigator who collated the laboratory results were blinded to each other's data. Trend of microalbuminuria was assessed from the change of ACR value within 6 hours of admission (ACR1) to the ACR value at 24 hours (ACR2) in both groups of patients. The difference of ACR2 from ACR1 (Δ ACR = ACR1 - ACR2) was calculated.

### Statistical Analysis

The Kolmogorov-Smirnov test was used to assess sample distributions. The results are presented as the median and 25th/75th percentiles (inter quartile range, IQR) (ACR data non-normally distributed). To compare two independent samples the Mann-Whitney U-test was used. The chi-square test was used to compare proportions. *P* < 0.05 was considered significant. Receiver operating characteristic (ROC) curves and the areas under the respective curve were calculated for ACR1, ACR2 and ΔACR. The discriminating powers of ACR variables (ACR1, ACR2, ΔACR) for the diagnosis of sepsis were assessed by the area under receiver operating characteristics (ROC) curves.

## Results

**Figure 1:**
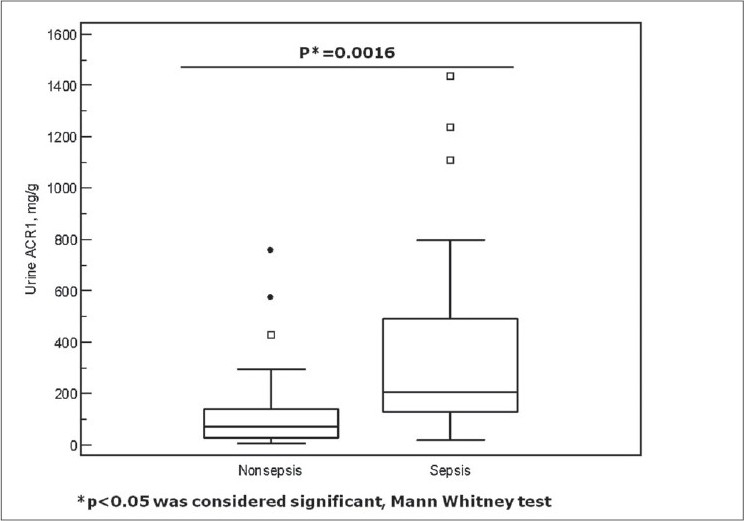
Box whisker plots comparing albumin:creatinine ratio (ACR) at ICU admission (ACR1), between patients with sepsis and without sepsis. Significant difference was found between the two groups (*P* = 0.0016). Open squares indicate outliers while the closed circles indicate extreme values. Similar difference was found in the ∆ ACR values of the patients with and without sepsis (*P* = 0.0048).

**Figure 2:**
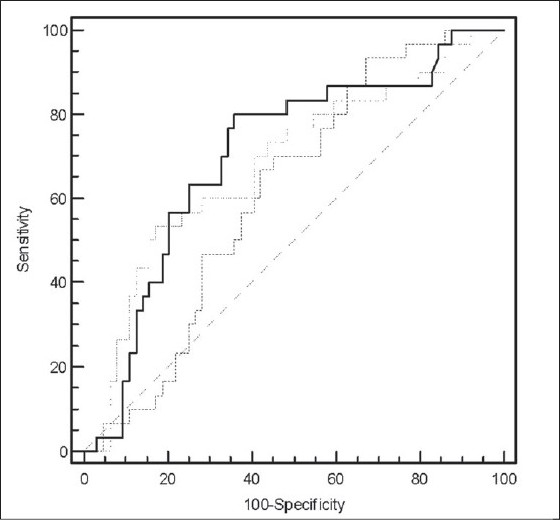
Comparison of Receiver Operating Characteristic (ROC) curves of ACR1 (straight line), ACR2 (dashed line) and the change of ACR2 from ACR1 (∆ ACR, dotted line) to discriminate between patients with and without sepsis admitted to intensive care unit. Of the three, ACR1 had the highest area under the curve.

**Figure 3:**
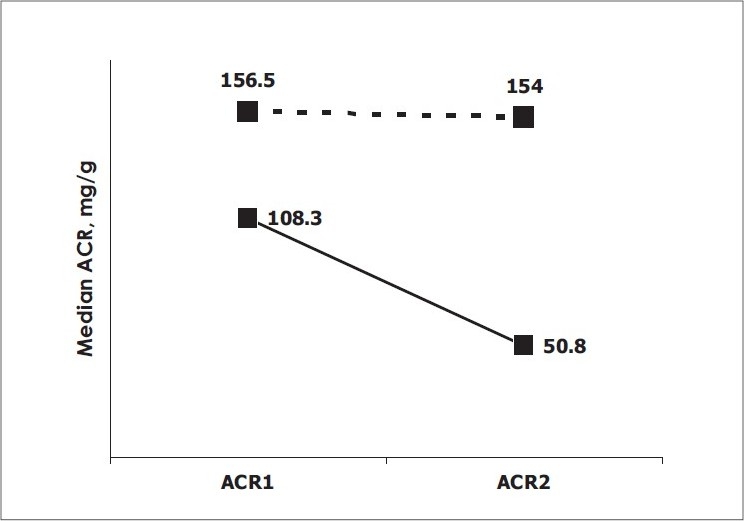
Change in urine albumin on intensive care unit admission (albumin-creatinine ratio [ACR] 1) and after 24 hours (ACR 2) in survivors (solid line) and nonsurvivors (dashed line) (Wilcoxon's signed rank test).

*Descriptive Characteristics of Patients:* Of 125 patients with an ICU stay of > 24 hours, who were recruited during the study period, a total of 94 patients were eligible for the study after exclusions. Patients with chronic kidney disease (n = 15), proteinuria due to renal and post renal disease (n = 6), hematuria (n = 6) and anuria (n = 4) were excluded. Patient demography and outcome variables are summarized in Table 1. Medical patients (94%) predominated in the study population. The primary reasons for admission to ICU were acute respiratory failure (COPD) (n = 18), gastrointestinal bleeding (n = 11), congestive heart failure (n = 5), acid base electrolyte disturbance (n = 5), and other causes (n = 55). Of the 94 patients, 30 (32%) were classified as sepsis patients (23 sepsis, 2 severe sepsis and 5 septic shock patients), the remaining 64 (68%) comprised the non-sepsis group (12 patients without SIRS, 52 with SIRS). Thirty seven percent had pre-existing diabetes mellitus and 47% had hypertension as per admission records. The study population was moderately sick with a median APACHE II score of 16. Thirteen (14%) of the 94 patients expired on the ICU. *Trend of ACR on admission and at 24 hours in all patients:* Of all the patients admitted to ICU, 78% had microalbuminuria within 6 hours of admission with a median ACR value of 125.6 (IQR 37.4 - 229.7) mg/g. At 24 hours of admission, microalbuminuria persisted in 67% of the patients. The median ACR2 fell by half to 62.6 (IQR 26.1 - 171.4) mg/g. The median delta change in ACR from within 6 hours to 24 hours of admission was 29.1 (IQR -7.3 to 103.0) mg/g. There was no significant difference in the median ACR values of male and female patients, or between medical and surgical patients either within 6 hours or at 24 hours of admission to ICU (data not shown). However, the median ACR2 was significantly higher amongst the nonsurvivors [154.0 (IQR 114.4 - 395.3) mg/g] when compared to the survivors [50.8 (IQR 21.6 - 144.7) mg/g] (*P* = 0.004). A higher ACR at 24 hours of admission portended a worse prognosis. *Trend of ACR on admission and at 24 hours in sepsis patients:* Microalbuminuria occurred in 87% of the sepsis patients and continued till 24 hours of admission in all but two patients. In the non-sepsis group, 73 % of the patients were admitted with microalbuminuria and it persisted till 24 hours in 61% of these patients. A comparison of demography for patients with and without sepsis yielded no significant statistical difference in either median age or the male/female ratio. Patients with diabetes mellitus were similarly distributed in both groups. Patients with hypertension were however higher in the sepsis group. The sepsis group was sicker (higher APACHE II), had a prolonged ICU stay and a significantly higher number of nonsurvivors [Table 2]. The degree of microalbuminuria within 6 hours of admission was significantly higher in patients with sepsis at a median ACR of 206.5 (IQR 129.7 - 506.1) mg/g in comparison to a median ACR of 76.4 (IQR 29 - 167.1) mg/g obtained for those without (*P* = 0.0016) [Figure 1]. At 24 hours, median ACR fell in both the groups. Although ACR2 at 96.3 (IQR 39.8 - 171.4) mg/g reflected significant microalbuminuria in the patients with sepsis there was no statistical difference in the ACR 2 values of the sepsis and non-sepsis groups (*P* = 0.08). The median delta ACR value was significantly greater in the sepsis patients [98.2 (IQR 12.4 - 213.1) mg/g] in comparison to the non-sepsis patients [11.7 (-34 to 64.1) mg/g] (*P* value: 0.0048). *Comparative diagnostic accuracy of urine albumin:creatinine ratio (ACR) variables for the differential diagnosis of sepsis after ICU admission:* For all patients, the area under the ROC curve (AUC) to distinguish sepsis was highest for ACR1 (0.702) followed by ACR2 (AUC 0.612) and ΔACR (AUC 0.681) [Figure 2]. Having identified ACR1 as the better discriminator, the sensitivity and specificity of ACR1 in diagnosing sepsis was calculated. At a cut off of 124 mg/g the sensitivity was 80% and specificity of 64%, positive predictive value (PPV) 51% and a negative predictive value (NPV) 87% [Table 3]. *Change in Urine Albumin following ICU admission compared with outcome.* A comparison between the survivors and nonsurvivors showed that the patients who died on the ICU had a significantly higher median APACHE II score, greater median duration of ICU stay and a significantly higher median ACR2 [Table 4]. While the change in the median levels of microalbuminuria from admission to 24 hours in the ICU was significant in the survivors, the median ACR for patients who died on the ICU remained unchanged 24 hours after ICU admission [Figure 3]. *Comparision of microalbuminuria with clinical outcome*: For all patients, ACR1 was significantly associated with duration of mechanical ventilation (*P* = 0.04). Both ACR1 (*P* = 0.007) and ACR2 (*P* < 0.001) were strongly correlated with APACHE II scores. *Comparative diagnostic accuracy of urine albumin-creatinine ratio and APACHE II scores for predicting ICU mortality:* For the entire population, the area under the ROC curves for prediction of mortality was highest for APACHE II (area under the curve 0.85), followed by ACR 2 (AUC 0.75), Δ ACR (AUC 0.61), and ACR 1 (AUC 0.57). There was no significant difference between the area under the curve for APACHE II and ACR2 (*P* = 0.3). To estimate the diagnostic accuracy of the urine albumin-creatinine ratio in the prediction of ICU mortality, the sensitivity and specificity were determined for an optimum cut-off level of ACR2 at 99.6 mg/g. At this value, ACR2 had a sensitivity of 85%, specificity of 68% with a NPV of 97% and PPV of 30% for the prediction of death.

**Table 1:** Patient demography, medical/surgical classification, median APACHE II score, duration of ICU stay, number of survivors and nonsurvivors

No. of patients	94
Median age, yrs (IQR)	63.5 (55–72)
Male (%): Female (%)	59 (62.8%): 35 (37.2%)
Medical (%): Surgical (%)	88 (93.6%): 6 (6.4%)
Median APACHE II (IQR)	16 (11–21)
Median duration of ICU stay, days (IQR)	4 (3–7)
Survivors (%)	81 (86.2%)
Non-survivors (%)	13 (13.8%)

**Table 2:** Comparing demographics, number of survivors, median APACHE II scores and median duration of ICU stay of patient groups with and without sepsis

	Nonsepsis group	Sepsis group	*P* value*
Number of patients	64	30	
Median age, yrs (IQR)	64 (57–71.5)	61.5 (52–72)	0.64
Males (%): Females (%)	39 (60.9%):	20 (66.7%):	0.75
	25 (29.1%)	10 (33.3%)	
Patients with Diabetes mellitus (%)	22 (34.4%)	13 (43.3%)	0.55
Patients with Hypertension (%)	24 (37.5%)	20 (66.7%)	0.02
Survivors (%):Non-survivors (%)	59 (92.2%):	22 (73.3%):	0.03
	5 (7.8%)	8 (26.7%)	
Med APACHE II score (IQR)	14 (11–20)	18 (13–26)	0.01
Median duration of ICU stay, days (IQR)	3 (2–4)	5 (3–10)	0.0025

* *P* < 0.05 was considered significant

**Table 3:** Receiver Operating Characteristic (ROC) curve analysis of ACR1, ACR2 and Δ ACR in differentiating patients with sepsis from those without for all patients (n = 94).

	ACR1	ACR2	Δ ACR
Cut Off (mg/g)	124	25.3	90.3
Area Under ROC Curve	0.702	0.612	0.681
Sensitivity	80%	93.3%	53.3%
Specificity	64.1%	32.8%	82.8%
Positive Predictive Value (PPV)	51.1%	39.4%	59.2%
Negative Predictive Value (NPV)	87.3%	91.3%	79.1%

**Table 4:** Comparing demographics, number of patients with diabetes, hypertension and sepsis, median APACHE II scores and median duration of ICU stay between patients who survived and those who died on the ICU.

	Survivors (n = 81)	Non survivors (n = 13)	*P* value
Median Age (IQR)	64 (56.8–72)	61 (48.8–70.3)	0.64
Males (%)	52 (64.2%)	7 (53.8%)	0.68
Patients with diabetes (%)	31 (38.3%)	4 (30.8%)	0.83
Patients with hypertension (%)	38 (46.9%)	6 (46.2%)	0.8
Patients with sepsis (%)	22 (27.2%)	8 (61.5%)	0.03
Median APACHE II score (IQR)	14 (11–19)	26 (21–28)	0.0001
ACR1, mg/g	108.3 (32.1–245.5)	156.5 (90.4–222.8)	0.41
ACR2, mg/g	50.8 (21.6–144.7)	154 (114.4–395.3)	0.004
Δ ACR, mg/g	30 (-3.3–129.2)	1.3 (-182.3–73.6)	0.22
Median duration of ICU stay, days (IQR)	3 (2–5)	8 (4.5–10.5)	0.005

*P < 0.05 was considered significant, Mann-Whitney U test.

## Discussion

Diagnosing sepsis early is vital for patient management and outcome but there is no reliable way of doing it yet. Culture of body fluids, the gold standard, are not always positive, and yield results not earlier than 24 h, which may be too late for administering targeted therapies. PCT is considered specific and sensitive in identifying systemic bacterial infections but increases in several non-infectious inflammatory conditions, absence of increase in localized infections, relatively high turnaround time of the LUMI test are some of its major limitations. A relatively less expensive alternative, CRP, is limited by a low specificity for the diagnosis of sepsis, slow induction time and lack of correlation with severity of disease.[26–28]

Microalbuminuria may serve as a means of indirectly quantifying these changes in systemic vascular permeability. Assay of the amount of albumin excreted in a random urine sample, expressed as ACR, is a simple, validated and reliable test.[29] Levels of microalbuminuria increase within hours of an inflammatory insult as compared to relatively delayed inductions of PCT and CRP.[30] Several studies in various groups of critically ill patients have unequivocally established microalbuminuria as a significant prognostic marker of morbidity and mortality in the ICU.[19]

Our study, conducted in a medical-surgical ICU, suggests a novel discriminatory role of microalbuminuria. On admission, microalbuminuria was found to be significantly elevated in patients with sepsis as compared to patients without (*P* = 0.0016). Later at 24 hours, ACR was significantly elevated in the non-survivors (*P* = 0.004). What could be the potential explanation for these observations? The pathophysiological cause of microalbuminuria in general is not known, but defects in both the glomerulus and the tubules have been implicated. In acute inflammation, microalbuminuria is surmised to be a result of the endothelial glomerular leak in the kidneys that is a manifestation of the systemic increases in capillary permeability, due to an intense inflammatory onslaught on the endothelium.[7,31] We postulate the possibility of inflammation induced defects in the glycocalyx layer of the endothelium being responsible for higher levels of microalbuminuria in sepsis. It has been shown that the glycocalyx of the fenestrated glomerular capillaries acts as a barrier to protein permeability and (enzyme) degradation of the layer increases the passage of albumin across the glomerulus.[32]

A possible explanation for the reductions of median levels after 24 hours of ICU admission could be the effect of therapeutic interventions on the attenuation of the inflammatory process and pacifying effects on the endothelium. We speculate that early targeted interventions may help in the preservation of the glycocalyx from further degradation that might mitigate increases in vascular permeability.[33] The same logic might also explain the observed decreases in the median Δ ACR levels being larger in patients with sepsis as compared to the patients without sepsis (*P* = 0.0048). One can therefore envisage the possible utility of microalbuminuria in monitoring the effect of therapy too. Spapen and De Gaudio's team have, in two independent studies, used microalbuminuria as a tool to document the effect of treatment of high doses of N-acetylcysteine, an antioxidant, and low dose hydrocortisone respectively, in severe clinical sepsis.[34,35]

To our knowledge, no previous study has attempted to evaluate the role of microalbuminuria in the diagnosis of sepsis. Though our findings suggest that ACR may not have a good discriminant value for the diagnosis of sepsis (PPV of 51%), its appeal lies in it being a noninvasive, inexpensive and ready-to-use bedside screening test to identify the patients with SIRS who do not have sepsis (NPV 87%). Furthermore, the findings of 80% sensitivity and 64% specificity of 6 hours ACR appears comparable to the reported mean percentage sensitivity of 85% and specificity of 83% of PCT, and 69% and 61%, respectively, of CRP,[27] in differentiating infected individuals from uninfected controls. The ACR test is regularly done in our hospital laboratory and results can be made available as early as 30 minutes. The ACR can also be estimated by the ICU nurses themselves, as a point-of-care test, within 15 min, as shown in Gosling *et al*'s study.[18]

Our study has a few limitations. Firstly, our study cohort was predominantly medical (>90%); and therefore it would not be appropriate to extrapolate the findings to a surgical ICU population. Secondly, while it is true that many conditions such as age (>40 yrs), smoking, diabetes mellitus and hypertension are independent causes of microalbuminuria in the general population,[36,37] these patients were included, since their exclusion would have made the study population less representative of the real life scenario. Moreover, by choosing a 3-4 times higher cut-off value of ACR (124 mg/g for the diagnosis of sepsis and 100 mg/g for mortality), the high NPV of the test could rule out most patients who had elevated ACR (by the conventional cut-off of 30 mg/g) due to confounders. The presence of diabetes or hypertension did not act as a confounding factor in the comparative analysis of patients; as such patients were similarly distributed in the two groups [Tables 2 and 4]. Hypertensives were higher in the sepsis category but again there was no significant difference in the ACR levels between patients with and without hypertension in this group [ACR1 (*P* = 0.07), ACR2 (*P* = 0.16), Mann Whitney test]. Critically ill patients with renal and post renal insufficiency were excluded from the study, which may be a limitation to the universal applicability of microalbuminuria as a diagnostic tool.

This study puts forth several possibilities for the potential application of urine albumin measurement in the critically ill. Urine ACR is significantly higher in the sepsis cohort in comparison to other systemic inflammatory diseases that probably indicates a distinct yet unknown pathophysiology. Serial monitoring of bedside urine albumin-creatinine measurement may potentially aid clinical assessment in the early identification of patients with sepsis that requires early targeted therapy. Another potential application may be in excluding patients at risk at 24 hours of admission. The 24 hours ACR assessment predicts ICU survival and may have the potential to monitor the efficacy of therapeutic interventions delivered, such as fluid resuscitation, appropriate antibiotics, vasopressors and inotropes that affect the endothelium.

Future large scale studies incorporating wider clinical categories and independent confounders of microalbuminuria are envisaged to evaluate the robustness of the ACR test. It will also be worthwhile to study the kinetics of microalbuminuria in different categories of SIRS and sepsis and to evaluate its utility on a “sepsis prediction panel” in conjunction with other biomarkers.

## Conclusion

Microalbuminuria is of common occurrence in a heterogeneous critically ill population. Patients without significant microalbuminuria during first six hours of ICU admission are less likely to have sepsis. At 24 hours, absence of elevated levels of microalbuminuria is strongly predictive of ICU survival, equivalent to the time-tested APACHE II scores. Microalbuminuria is an inexpensive and rapid diagnostic tool; serial measurements may prove a useful aid in the clinical assessment of critically ill patients at risk of worse prognosis, even in resource poor areas.
